# Significant increase in the secretion of extracellular vesicles and antibiotics resistance from methicillin-resistant *Staphylococcus aureus* induced by ampicillin stress

**DOI:** 10.1038/s41598-020-78121-8

**Published:** 2020-12-03

**Authors:** Si Won Kim, Jong-Su Seo, Seong Bin Park, Ae Rin Lee, Jung Seok Lee, Jae Wook Jung, Jin Hong Chun, Jassy Mary S. Lazarte, Jaesung Kim, Jong-Hwan Kim, Jong-Wook Song, Chris Franco, Wei Zhang, Min Woo Ha, Seung-Mann Paek, Myunghwan Jung, Tae Sung Jung

**Affiliations:** 1 Laboratory of Aquatic Animal Diseases, College of Veterinary Medicine, Research Institute of Natural Science, Gyeongsang National University, Jinju, 52828 Republic of Korea; 2 Department of Environmental Toxicology and Chemistry, Environmental Chemistry Research Center, Korea Institute of Toxicology Gyeongnam, Jinju, 52834 Republic of Korea; 3 Coastal Research and Extension Center, Mississippi State University, Mississippi State, MS 39567 USA; 4 Centre for Marine Bioproducts Development, College of Medicine and Public Health, Flinders University, Bedford Park, Adelaide, SA 5042 Australia; 5 College of Pharmacy and Research Institute of Pharmaceutical Sciences, Gyeongsang National University, Jinju, 52828 Republic of Korea; 6 Department of Microbiology, Research Institute of Life Sciences, College of Medicine, Gyeongsang National University,, Jinju, 52727 Republic of Korea

**Keywords:** Antibiotics, Antimicrobial resistance

## Abstract

Extracellular vesicles (EVs) containing specific cargo molecules from the cell of origin are naturally secreted from bacteria. EVs play significant roles in protecting the bacterium, which can contribute to their survival in the presence of antibiotics. Herein, we isolated EVs from methicillin-resistant *Staphylococcus aureus* (MRSA) in an environment with or without stressor by adding ampicillin at a lower concentration than the minimum inhibitory concentration (MIC). We investigated whether EVs from MRSA under stress condition or normal condition could defend susceptible bacteria in the presence of several β-lactam antibiotics, and directly degrade the antibiotics. A comparative proteomic approach was carried out in both types of EVs to investigate β-lactam resistant determinants. The secretion of EVs from MRSA under antibiotic stressed conditions was increased by 22.4-fold compared with that of EVs without stress. Proteins related to the degradation of β-lactam antibiotics were abundant in EVs released from the stressed condition. Taken together, the present data reveal that EVs from MRSA play a crucial role in the survival of β-lactam susceptible bacteria by acting as the first line of defense against β-lactam antibiotics, and antibiotic stress leads to release EVs with high defense activity.

## Introduction

Although the discovery of antibiotics has prolonged human life and helped develop a variety of health-related technologies, the overuse of antibiotics has led to the emergence of "superbugs" as bacteria have evolved the means of resisting traditional antibiotics. The increase in superbug strains is believed to be a serious threat facing public health around the world. Damage caused by antimicrobial resistance is expected to reach a total gross domestic product (GDP) loss of $ 100 trillion worldwide by 2050, at which point it could kill 10 million people annually^1^. To combat the spread of superbugs globally, it is important to understand all possible bacterial protection mechanisms against antibiotics.

The multidrug-resistant (MDR) pathogenic bacterium, as the main cause of recurrent opportunistic infections, is methicillin-resistant *Staphylococcus aureus* (MRSA). Since MRSA was reported in 1961^2^, the incidence of this strain has spread worldwide. MRSA is a major Gram-positive bacterial pathogen that causes a range of illnesses including pneumonia, meningitis, osteomyelitis, endocarditis, and septicemia, which leads to high mortality and is expensive to treat^3^. Numerous cases of infections due to MRSA have been reported in companion, diverse domesticated, and livestock animals^4,5^. The transmission between humans and animals indicated that both directions of humanosis and zoonosis are possible^5,6^. Therefore, emerging MRSA infections are no longer primarily a human healthcare-related threat, but a global community-associated problem.

Bacteria secret proteins, polysaccharides and diverse molecules to their extracellular milieu to communicate and to coordinate population behaviors^7,8^. Among them, extracellular vesicles (EVs) are known to possess diverse cellular factors and have a variety of functions to aid bacteria survival^7–11^. EVs are defined as spherical, and bilayered proteolipids which form lumen-containing spheres with an average diameter of 20–200 nm, and composed of proteins from various cellular origins, unique lipids, enzymes, toxins and nucleic acids^7,8,12,13^. Packed within vesicles, the cargo molecules can be transported over long distances away from dilution and degradation, which might explain the effective interaction among bacteria^14^. These vesicles have been elucidated to play diverse roles^7–9,12,15^, thus EVs are considered the powerful intercellular and interspecies ‘communicasomes’ in the microbial ecosystem.

Although several studies suggested that EVs can defend the bacteria against the effects of several antibiotics by acting as decoys^9,16^, either through horizontal transfer of antibiotic resistance genes^15,17^, or degradation/sequestration of antibiotics^7,9,18,19^, the mechanism of EVs against antibiotics remain uncharacterized. Previous studies have reported that exposure to some physiological or environmental stressors such as antibiotic treatment, oxidative stress, and temperature influenced the level of vesicles secretion, and stressors could cause alterations in the composition of vesicles^11,20,21^. Based on these studies, we hypothesize that a physiological stressor such as sub-lethal antibiotics can trigger bacteria secreting EVs with significant changes in the proteome to adapt and survive in the antibiotic stressed environment.

In the present study, we compared the protein constituents of EVs from MRSA cultured under normal conditions (EV_Nor_) and cultured in the presence of sub-lethal concentrations of ampicillin (EV_Strs_). The results implicated that EV_Strs_, which have more proteins that can degrade antibiotics than EV_Nor_, can offer protection to the β-lactam-susceptible *S. aureus* against lethal β-lactam antibiotic concentrations better than EV_Nor_.

## Results

### Physical characterization of EVs

**Figure 1:**
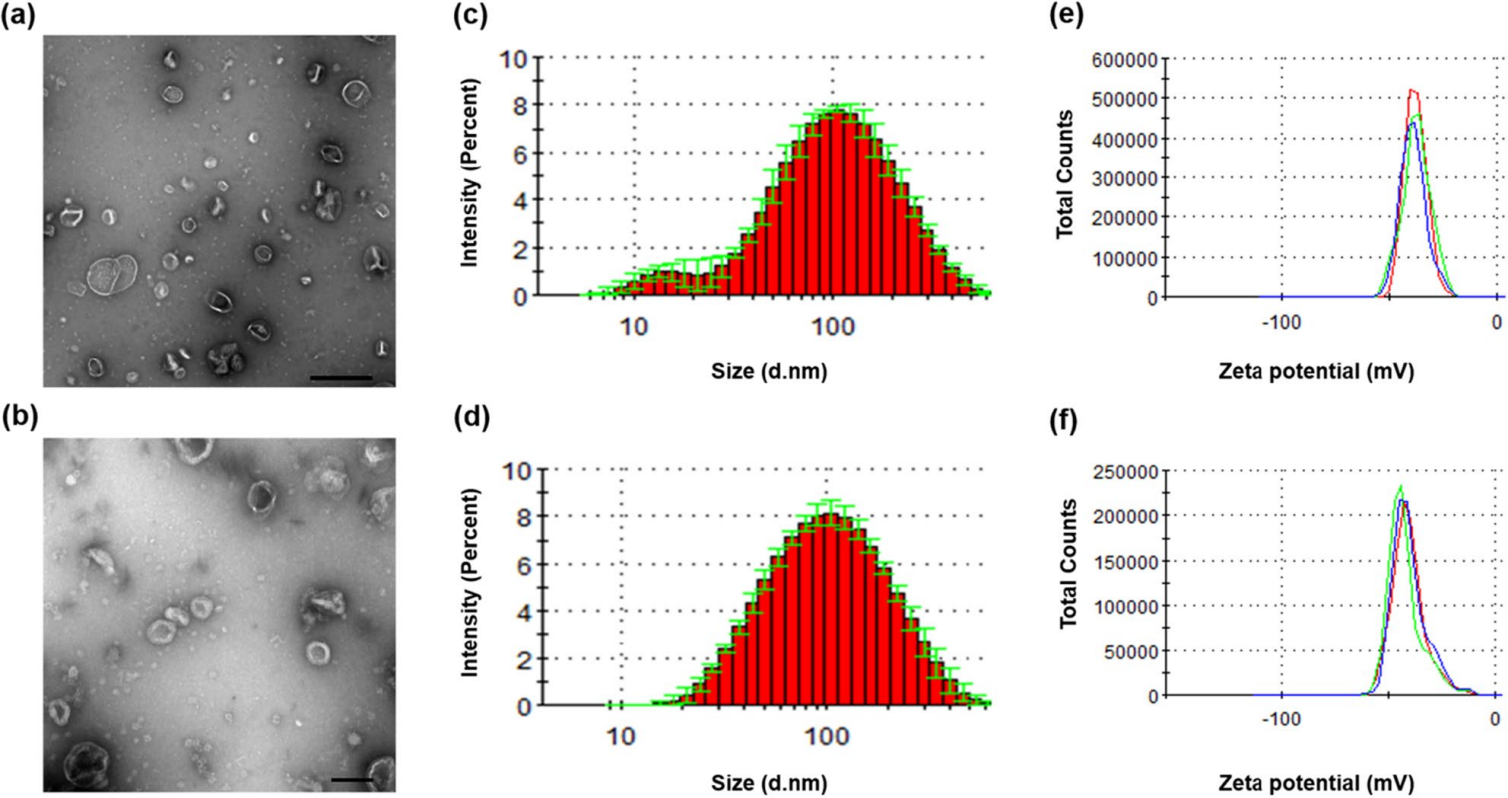
Physical characterizations of EVs derived from stressed and normal ST692 cells. TEM image of EVs derived from stressed ST692 (**a**) (scale bar: 500 nm) and normal ST692 (**b**) (scale bar: 100 nm) cells. The size distribution EVs released from stressed ST692 (**c**) and normal ST692 (**d**) cells. Three independent analyses were performed; means are shown with ± standard deviation (error bars) of the percentage intensity. The zeta potential of EVs from stressed ST692 (**e**) and normal ST692 (**f**) cells.

EVs of MRSA ST692 cells were isolated and designated as EV_Nor_ and EV_Strs_ with respect to the culture conditions. Transmission electron microscopy (TEM) analysis showed bi-layered spherical EVs (Fig. 1a,b). Dynamic light scattering (DLS) revealed the average diameter of EV_Strs_ (78.22 ± 0.81 nm) and EV_Nor_ (86.84 ± 0.25 nm) (Fig. 1c,d, and see Supplementary Table S1 online). EV_Strs_ has more vesicles of 10–20 nm size than EV_Nor_, but the size distribution except for 10–20 nm is almost identical with EV_Nor_. Their polydispersity index (PDI) were measured below 0.3, indicating that the arrangements were monodispersed (Supplementary Table S1). Their zeta potentials were more negative than -30 mV, implying that there were no considerable differences in the cohesion of the vesicles (Fig. 1e,f, and Supplementary Table S1).

### Vesiculation enhances upon physiological stress

**Figure 2:**
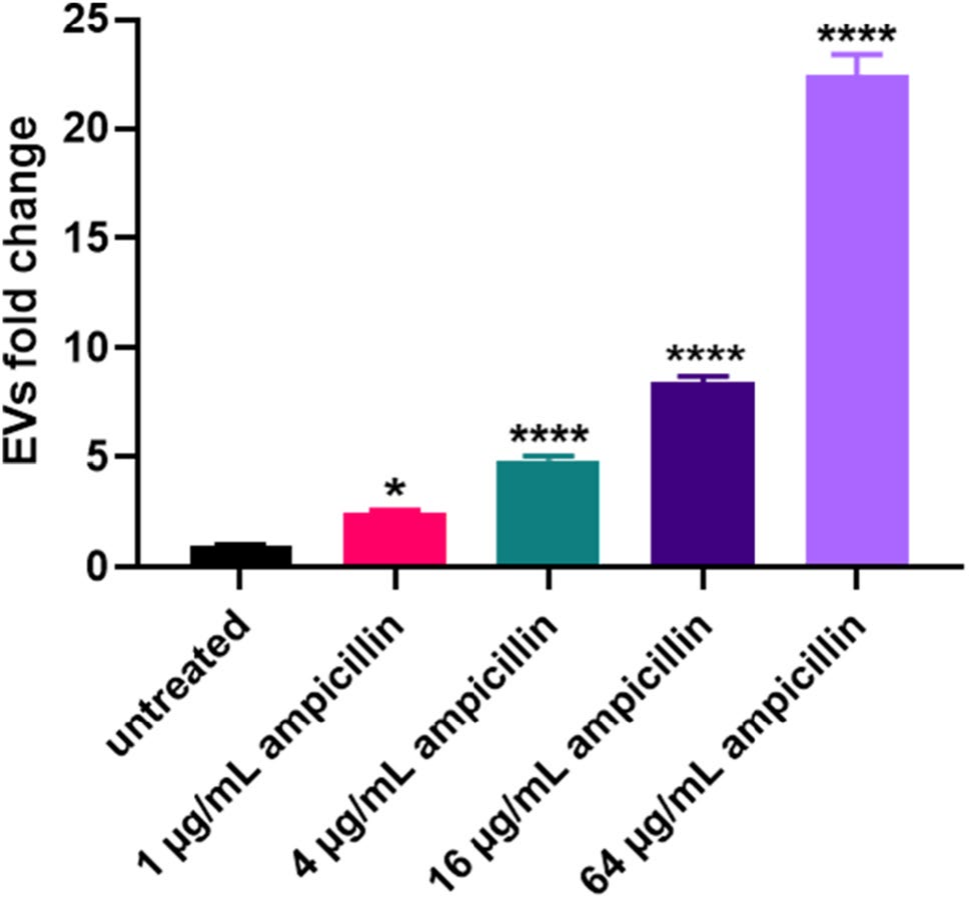
Induction of EVs production by treatments with stressors. (**a**) EVs were purified and quantified from cultures of ST692 cells treated with 64, 16, 4, 1, or 0 μg/mL ampicillin. EVs yields were averaged and normalized to untreated controls to adjust fold change. One way ANOVA was used for analyses and data were presented as mean ± standard deviations (SD). *P < 0.05, ****P < 0.0001.

To understand further how ampicillin stress affects EVs production, ST692 was exposed to incremental concentrations of ampicillin and each of the respective EVs was purified (Fig. 2). The quantity of secreted EVs increased dose-dependently according to the amount of ampicillin added to the culture of ST692 cells. In particular, the production of EV_Strs_ (64 μg/mL of ampicillin treated) increased by 22.4-fold compared to EV_Nor_, the untreated control. Even when only 1 μg/mL of ampicillin was added, the yield was 2.5-fold higher than EV_Nor_.

### EVs defend β-lactam susceptible *S. aureus* cells against β-lactam antibiotics

**Figure 3:**
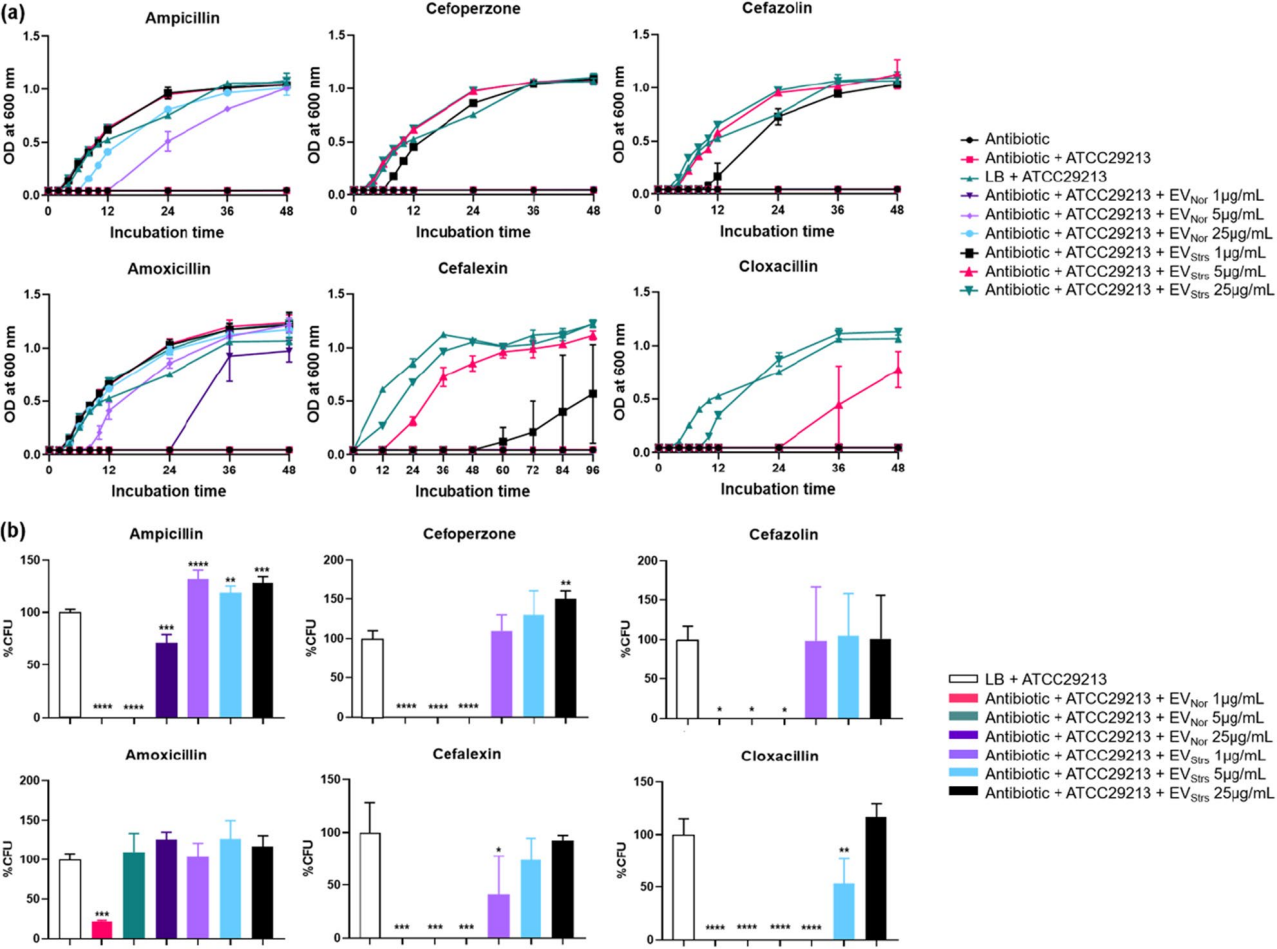
EVs from methicillin-resistant *Staphylococcus aureus* (MRSA) can defend β-lactam-susceptible *S. aureus* and fully protect them from β-lactam antibiotic-induced growth inhibition. (**a**) Representative growth profiles of β-lactam-susceptible *S. aureus* cells in the presence of growth-inhibiting concentrations of β-lactam antibiotics. The growth-inhibiting concentrations of antibiotics were: ampicillin, 40 μg/mL; cefoperazone, 8 μg/mL; cefazolin, 1.25 μg/mL; amoxicillin, 40 μg/mL; cefalexin, 4 μg/mL; and cloxacillin, 1.25 μg/mL. The data were presented as means and SEMs of at least three independent experiments. (**b**) The survival percentages of β-lactam-susceptible *S. aureus* cells in the presence of the above-listed growth-inhibiting concentrations of antibiotics and EVs from stress condition or normal condition were calculated by bacterial counts of cultures at a certain time points (ampicillin, 12 h; cefoperazone, 12 h; cefazolin, 24 h; amoxicillin, 36 h; cefalexin, 96 h; and cloxacillin, 48 h). CFU of *S. aureus* cells in medium without any antibiotics were used as a positive control and taken as 100%, and the corresponding CFU of samples was computed. The data were presented as means and SEMs of three independent experiments. *P < 0.05, **P < 0.01, ***P < 0.001, ****P < 0.0001.

Minimum inhibitory concentrations (MICs) of the MRSA strain ST692 and the susceptible bacterium *S. aureus* ATCC29213 were measured to determine whether the different concentrations of EV_Strs_ and EV_Nor_ from ST692 could protect *S. aureus* ATCC29213 against several antibiotics (Table 1). The growth kinetics presented in Fig. 3a showed that EVs can protect susceptible bacteria against each antibiotic at a higher concentration than the MIC of *S. aureus* ATCC29213. For the six β-lactam antibiotics, EV_Strs_ dose-dependently protected *S. aureus* ATCC29213, allowing it to tolerate antibiotic exposures above the MICs. EV_Nor_ also dose-dependently defended ATCC29213 against ampicillin, and amoxicillin, but did not protect the susceptible bacteria from the other antibiotics tested over the incubation time. Since the equivalent amount of EV_Strs_ protects susceptible bacteria from antibiotics more strongly than that of EV_Nor_, the susceptible strains grew much faster in the EV_Strs_ group. One microgram per milliliter of EV_Strs_ appears to be more protective than 25 μg/mL of EV_Nor_. However, neither EV_Strs_ nor EV_Nor_ protected the susceptible bacteria against antibiotics other than the six mentioned above (cefotaxime, imipenem, methicillin, chloramphenicol, gentamicin, kanamycin, streptomycin, and tetracycline) (data not shown).

**Table 1:** The MIC of several antibiotics against the β-lactam-susceptible *Staphylococcus aureus* ATCC29213 and β-lactam-resistant *S. aureus* ST692.

Class	Antibiotics	MIC (μg/mL)	
		ATCC29213	ST692
β-Lactams	Ampicillin	2	256
	Amoxicillin	4	256
	Cefalexin	1	32
	Cefazolin	0.25	32
	Cefoperazone	4	64
	Cefotaxime	1	32
	Cloxacillin	0.25	< 0.5
	Imipenem	8	> 1024
	Methicillin	1	32
Chloramphenicol	Chloramphenicol	8	16
Aminoglycosides	Gentamicin	4	4
	Kanamycin	16	32
	Streptomycin	32	> 1024
Tetracyclines	Tetracycline	0.5	64

*MIC* minimum inhibitory concentration.

### Bacterial protection percentage of EV_Strs_ and EV_Nor_ from antibiotics

To determine the degree of bacterial protection of each EV against antibiotics, we performed quantitative plate assays based on growth kinetics (Fig. 3b). Notably, the viability of the susceptible cells inoculated with EV_Strs_ at 25 μg/mL in the presence of respective antibiotics showed no loss in viability compared to the culture of susceptible cells without antibiotics. In contrast, the viability of the susceptible cells with EV_Nor_ at 25 μg/mL in the presence of cefoperazone, cefazolin, cefalexin, and cloxacillin showed no viability, which can be explained by the rapid killing by the respective antibiotic concentrations.

### Specific molecules of EVs are important for the protection of bacteria

After the growth curve experiment, all samples were plated on TSA agar with or without respective antibiotics in the same concentration as was used in the growth curve experiment (see Supplementary Fig. S1 online). All samples that grew in the above experiment were grown in TSA agar but not in TSA agar with respective antibiotics. These results suggested that the enhanced survival rates were not due to a mutation of the β-lactam resistant genetic materials of EVs transferred to susceptible *S. aureus*, but rather from the molecules owned by EVs that protected the bacteria from the β-lactam antibiotic environment. In addition, colonies grown in TSA agar were identified as *S. aureus* at the species level using MALDI-TOF MS (data not shown).

### EVs protect different genera of Gram-negative bacteria against ampicillin

**Figure 4:**
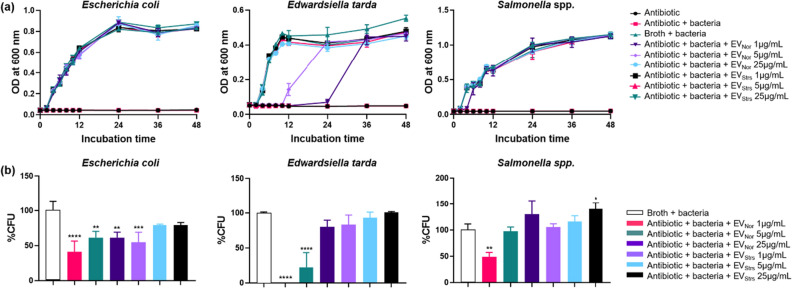
EVs from methicillin-resistant *Staphylococcus aureus* (MRSA) can protect other bacterial species from ampicillin-induced growth inhibition. (**a**) Representative growth profiles of *Escherichia coli* (RC85), *Edwardsiella tarda* (ED45), and *Salmonella* spp. (Sal26B) cells in the presence of a growth-inhibiting concentration of ampicillin (30 μg/mL) plus increasing amounts of EVs from stress conditions or normal conditions. The data were presented as means and SEMs of at least three independent experiments. (**b**) The survival percentages of RC85, ED45, and Sal26B cells were calculated by counting CFUs at specific time points (12 h) from cultures grown with 30 μg/mL ampicillin and increasing quantities of EVs from stress condition or normal condition. CFU of respective bacterial cells in medium without ampicillin was used as a positive control and taken as 100%, and the corresponding CFU of samples was calculated. The data were presented as means and SEMs of three independent experiments. *P < 0.05, **P < 0.01, ***P < 0.001, ****P < 0.0001.

To determine whether the EVs isolated from the Gram-positive MRSA can protect Gram-negative bacteria, which belong to different genera, growth curve profiles in the presence of growth-inhibiting concentrations of ampicillin and qualitative plate assays were carried out. The MICs of ampicillin against *Escherichia coli* (RC85), *Edwardsiella tarda* (ED45), and *Salmonella* spp. (Sal26B) were 8 μg/mL, 4 μg/mL, and 8 μg/mL, respectively^9^. Both EV_Strs_ and EV_Nor_ defended RC85, ED45, and Sal26B against ampicillin (Fig. 4a). ED45 cells treated with 5 μg/mL EV_Nor_ exhibited growth at 12 h, whereas both RC85 and Sal26B cells supplemented with 5 μg/mL exhibited growth at 2 h. When EV_Nor_ 1 μg/mL was added to each strain, RC85 exhibited growth at 2 h, ED45 at 24 h, and Sal26B at 4 h. Since the MIC of ED45 was lower than that of RC85 or Sal26B, the protective effect caused by EVs molecules appears to be slower. Quantitative plate assays were investigated by counting the CFU based on growth kinetics (Fig. 4b). Both EV_Strs_ and EV_Nor_ dose-dependently protected each susceptible strains against the bactericidal effect of ampicillin.

### EVs enable degradation of β-lactam antibiotics

**Figure 5:**
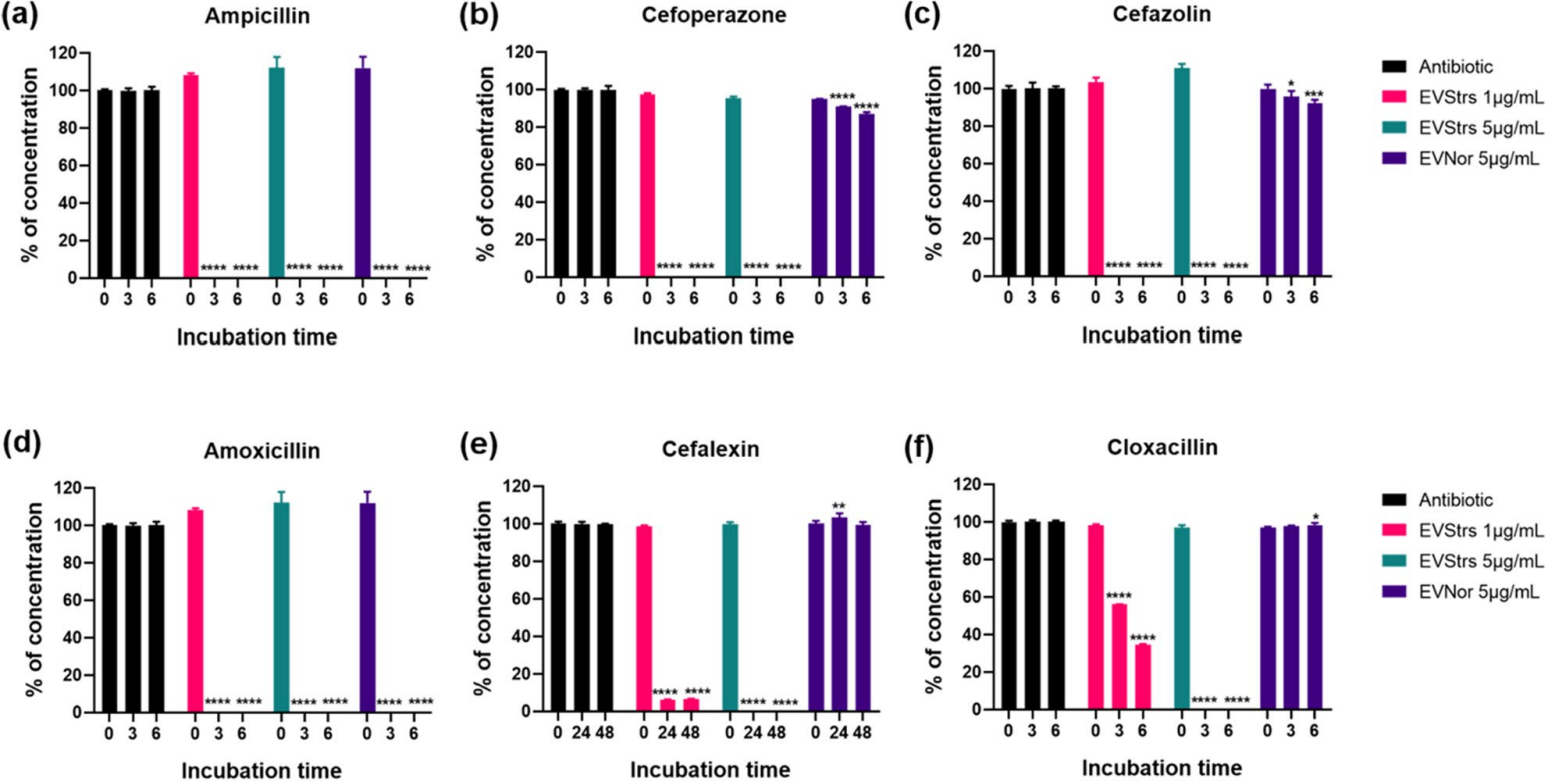
LC-QQQ-based evaluation of the concentration of β-lactam antibiotics following incubation with different doses of EVs from stress conditions or normal conditions in a cell-free system. The original concentrations were as follows: ampicillin, 40 μg/mL (**a**); cefoperazone, 8 μg/mL (**b**); cefazolin, 1.25 μg/mL (**c**); amoxicillin, 40 μg/mL (**d**); cefalexin, 4 μg/mL (**e**); cloxacillin, 1.25 μg/mL (**f**). One microgram per milliliter or 5 μg/mL of EVs from stress condition or 5 μg/mL of EVs from a normal conditions in sterilized PBS were mixed with respective antibiotics. Each of the antibiotic without EVs was averaged and normalized as 100%, and the corresponding concentrations of antibiotics in samples treated with EVs were analyzed. The concentrations of antibiotics were registered at 3-h intervals for 6 h (**a**–**d**,**f**) or 24-h intervals for 48 h (**e**) in triplicate. Bars indicate standard deviations. *P < 0.05, **P < 0.01, ***P < 0.001, ****P < 0.0001.

**Figure 6:**
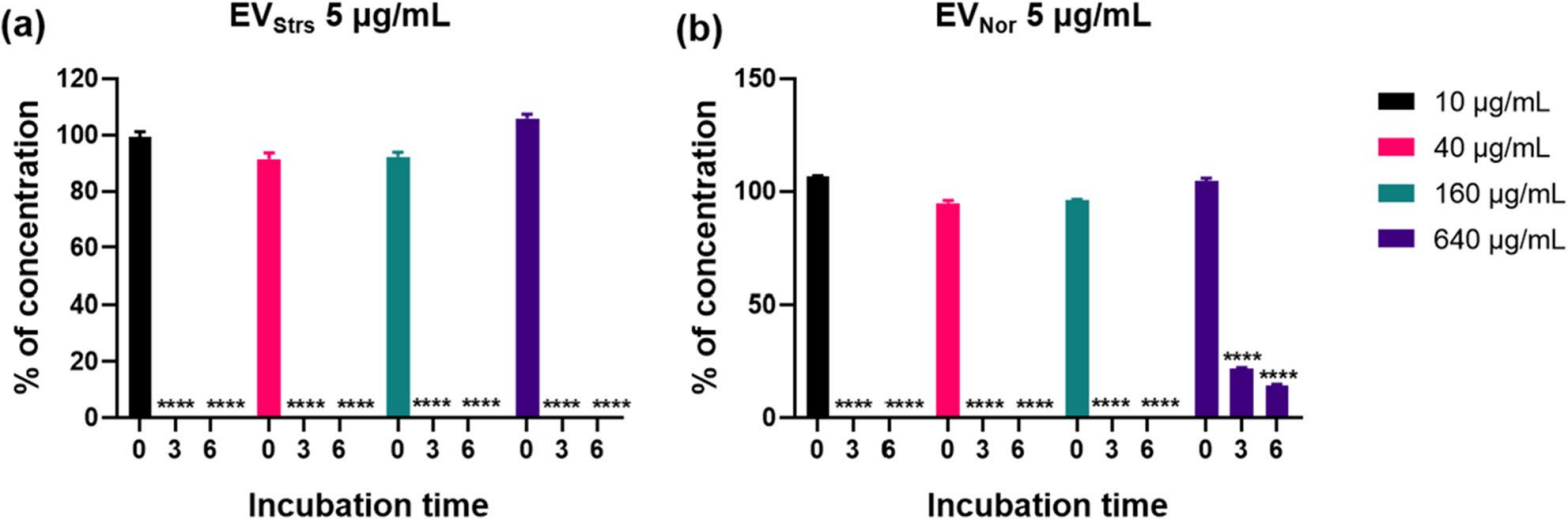
Consumptive ampicillin titration of EVs from stress condition and normal condition using LC-QQQ-based assessment in a cell-free system. The concentration of each EVs was fixed at 5 μg/mL, and the concentration of ampicillin was changed to 10, 40, 160, or 640 μg/mL. Ampicillin concentration was determined at an interval of 3 h up to 6 h. Ampicillin without EVs was used as a positive control and taken as 100%, and the corresponding concentrations of ampicillin in samples treated with EV_Strs_ and EV_Nor_ were analyzed. ****P < 0.0001.

To explain the reason why EVs were capable of protecting bacteria in the β-lactam antibiotic environment, LC-ESI-QQQ analysis was carried out to measure concentrations of antibiotics after treatment of EVs in the presence of antibiotics in a cell-free system (Fig. 5). The concentration of six antibiotics was dramatically decreased in samples treated with 5 μg/mL of EV_Strs_ for 3 h (five antibiotics except for cefalexin) or 24 h (cefalexin) compared with respective antibiotics without EVs. Samples treated with 1 μg/mL of EV_Strs_ could hydrolyze six antibiotics, but the capacity appears to be lower than that of 5 μg/mL. Moreover, 5 μg/mL of EV_Nor_ also completely decomposed ampicillin and amoxicillin in 3 h, but the activity against cefoperazone and cefazolin was very weak, and against cefalexin and cloxacillin was not detected. The extent of the capacity of EV_Strs_ and EV_Nor_ to hydrolyze ampicillin for 6 h was compared by increasing the dose of ampicillin after EVs concentrations were fixed to a certain amount (Fig. 6). EV_Strs_ degraded 640 μg/mL of ampicillin in 3 h, but EV_Nor_ could not degrade the equivalent concentration of antibiotics for 6 h.

### Proteomic characterization of EV_Strs_ and EV_Nor_

**Figure 7:**
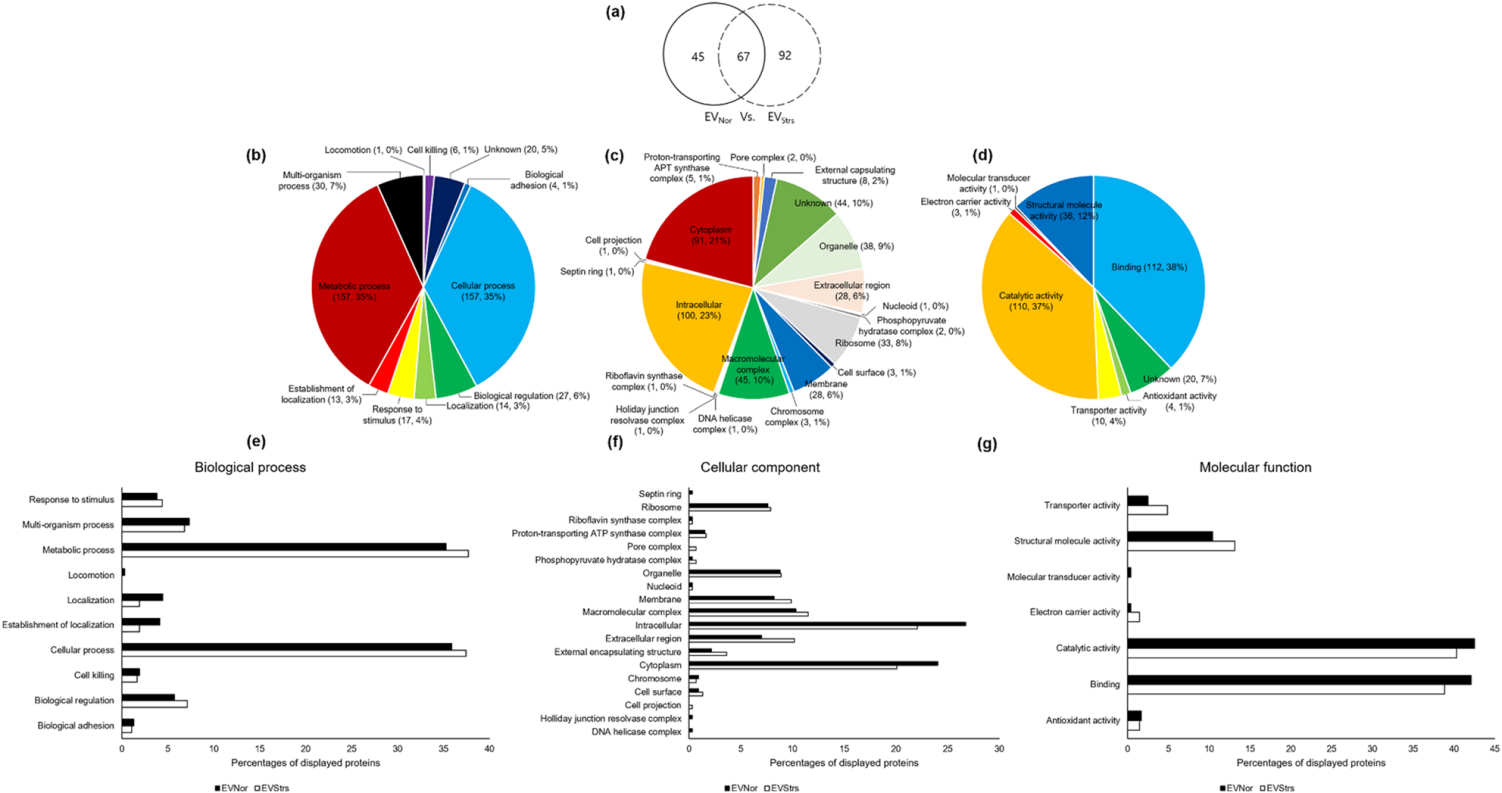
Venn diagrams show the entire proteins identified from EV_Nor_ and EV_Strs_ searched against the uni_bacteria database while the bar graphs classify the proteins which showed differential expression in EV_Nor_ and EV_Strs_. (**a**) A total of 204 proteins were established in EV_Nor_ (112 Proteins) and EV_Strs_ (159 Proteins) together. Entire proteins were classified depending on the related biological process (**b**), cellular component (**c**), and molecular function (**d**). The percentages of displayed EV_Nor_ (black) and EV_Strs_ (white) proteins were compared with respect to the consistent biological process (**e**), cellular component (**f**), and molecular function (**g**).

We compared the protein constituents of EV_Strs_ and EV_Nor_ using LC–MS/MS analysis because delineation of the biological role of the protein components of EVs is important in understanding their relevance to antibiotic resistance. A total of 204 proteins were identified using the uni_bacteria database (Fig. 7a, See Dataset 1 online). EV_Strs_ alone has 159 proteins, of which 67 proteins overlapped with EV_Nor_. All proteins possessed by EV_Strs_ and EV_Nor_ were classified according to these categories: relevant biological processes, cellular components, and molecular functions (Fig. 7b–d). Each of the proteins of EV_Strs_ and EV_Nor_ was also classified with the same categories (Fig. 7e–g). The introduction of the LC–MS/MS data into the *Staphylococcus aureus* USA300 strain database revealed that there are several different proteins identified which are not included in the uni_bacteria database. So we re-examined the proteins under the categories, biological processes, cellular components, and molecular functions of both EV_Strs_ and EV_Nor_, separately and combined (see Supplementary Fig. S2 and Dataset 2 online). The results obtained here implied that the majority of the protein composition of EVs was altered and some of the proteins are more abundant which might be the result of the reaction of the host bacteria to the exposure of sub-MICs of ampicillin.

### Comparative analysis of EVs protein concerned with resistance to a β-lactam antibiotic

Among the EVs proteins analyzed from the two databases (Dataset 1 and Dataset 2), we examined the candidate proteins related to the consumption of β-lactam antibiotics (Table 2). The β-lactamase proteins encoded by *blaZ* and *SA1529 *and native PBPs such as PBP1, -2, -3 were upregulated when compared to the protein compositions of EV_Strs_ versus EV_Nor_.

**Table 2:** Quantitative candidate protein profiling involved in the consumption of β-lactam antibiotics by EVs.

Identified proteins	Accession number	Alternate ID	Quantitative value (normalized total spectra)	
			EV_Nor_	EV_Strs_
Beta-lactamase	BLAC_STAAU	blaZ	111.86	607.48
UPF0173 metal-dependent hydrolase	Y1529_STAAN (+ 12)	SA1529	0	4.4352
Penicillin-binding protein 1	A0A0H2XJZ5_STAA3	pbpA	0	25.135
Penicillin binding protein 2	A0A0H2XI32_STAA3	pbp2	16.733	119.2
Penicillin-binding protein 3	A0A0H2XJ39_STAA3	pbp3	12.932	122.17

### β-lactamase content in EVs was regulated by ampicillin stress

**Figure 8:**
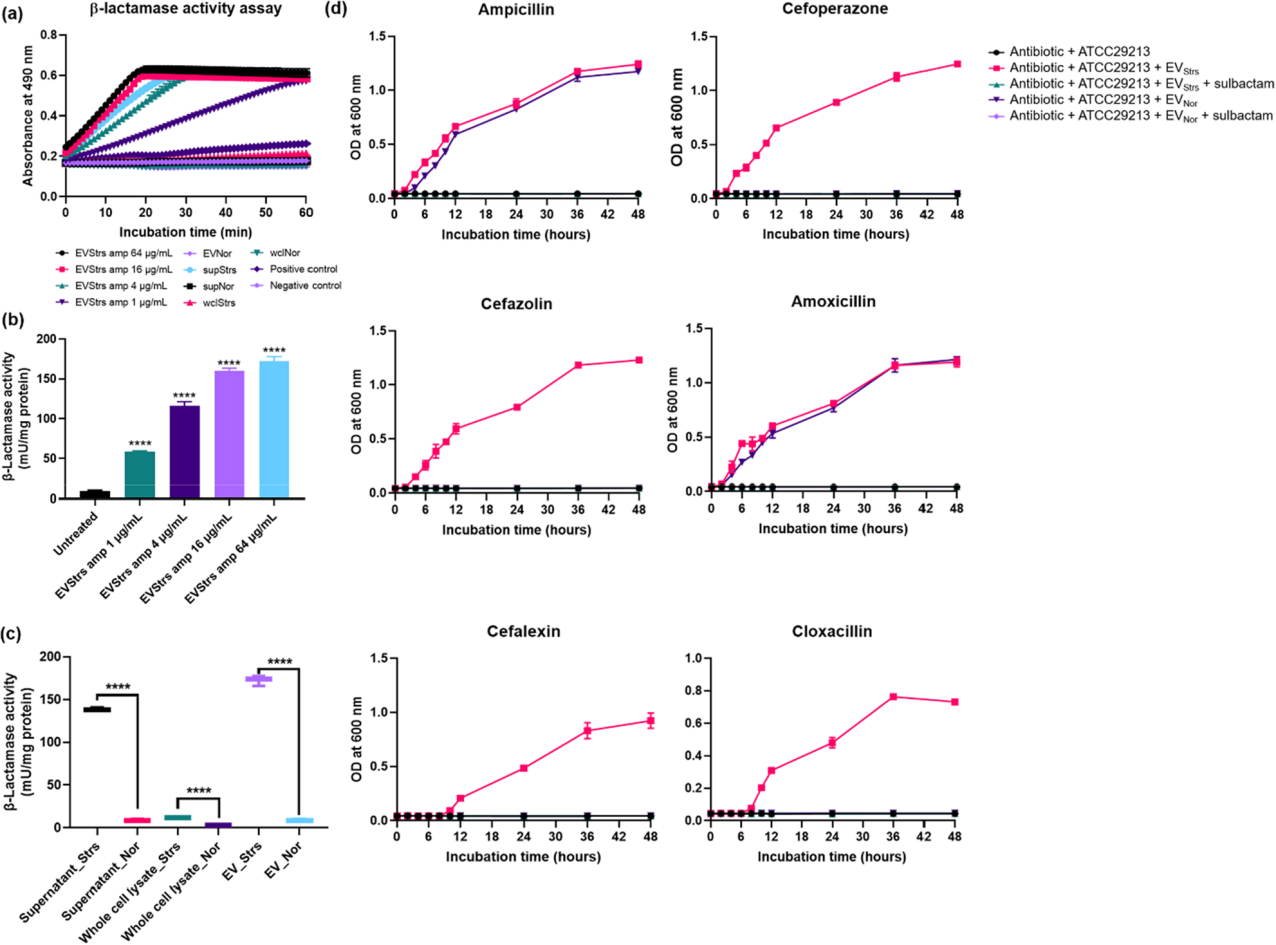
Comparison and analysis of β-lactamase activity of supernatant (sup), whole-cell lysate (wcl), and EVs isolated from stressed strain and normal strain. ‘S’ means stressed and ‘N’ means normal. (**a**) β-Lactamase activity profiles of respective samples, as examined by measuring absorbance at 490 nm in kinetic mode. (**b**) EVs from cultures of ST692 cells treated with 64, 16, 4, 1 μg/mL ampicillin, or untreated were assayed for β-lactamase activity. (**c**) β-Lactamase activities expressed per milligram of protein. (**d**) The effects of the β-lactamase inhibitor, sulbactam, were examined by growth curve experiments of β-lactam-susceptible *Staphylococcus aureus* treated with six antibiotics (ampicillin, 40 μg/mL; cefoperazone, 8 μg/mL; cefazolin, 1.25 μg/mL; amoxicillin, 40 μg/mL; cefalexin, 4 μg/mL; cloxacillin 1.25 μg/mL) plus sulbactam in the presence of 25 μg/mL EV_Strs_ or EV_Nor_. All data were presented as means and SEMs of three independent experiments. ****P < 0.0001.

To further elaborate on the relevance of ampicillin stress in the total production of β-lactamase in EVs of the host bacteria, we then analyzed the β-lactamase activity associated with the degradation of various β-lactam antibiotics. All samples of EVs from cultures treated with increasing concentrations of ampicillin showed higher absorbance than the positive control, other samples had higher absorbance than negative control but lower than the positive control (Fig. 8a). To quantify the β-lactamase activity of EVs was presented as milliunit per milligram of protein (Fig. 8b). The activity of EVs isolated from bacteria exposed to increasing concentration of ampicillin in sub-MICs increased gradually, wherein, EVs treated with 1 μg/mL exhibited more β-lactamase activity than corresponding EV_Nor_, by 6.5-fold. When comparing the samples with β-lactamase activities per mg, the EV_Strs_ (treated with 64 μg/mL of ampicillin) was the highest, followed by the supernatant from stress condition (Fig. 8c). All of the samples subjected to ampicillin stress showed significantly increased levels as opposed to those in the stress-free environment, specifically in the supernatant by 15.8-fold, whole-cell lysate by 4.0-fold, and EVs by 19.1-fold.

### The function of EVs mixed with a β-lactamase inhibitor

To determine whether β-lactamase is involved in the antibiotic degradation capacity of EVs, we observed the growth kinetics after the treatment of EVs and β-lactamase inhibitors (sulbactam) in ATCC29213 cells in the respective antibiotic environment (Fig. 8d). EV_Strs_ degraded each β-lactam antibiotics more than EV_Nor_, allowing susceptible cells to grow in an antibiotic environment, but no susceptible cells grew within 48 h in the respective EVs added with sulbactam.

### Ampicillin stress affects β-lactam antibiotic tolerance of bacteria

The MICs of each antibiotic against stressed and normal ST692 cells were compared (Table 3). Treatment of ampicillin lower than MICs (64 μg/mL) increased the MICs from 2 to 4 times for β-lactam antibiotics except for cefotaxime, imipenem, and methicillin, but when the stressed ST692 cells were sub-cultured in antibiotic-free agar they reverted to their normal susceptibility to a β-lactam antibiotic. The MICs of stressed ST692 cells showed no change in all other antibiotics except for β-lactam antibiotics.

**Table 3:** Alteration in MIC of several antibiotics against *Staphylococcus aureus* ST692 and ST692 stressed on ampicillin.

Class	Antibiotics	MIC (μg/mL)		Fold increase in MIC (stress/normal)
		Normal ST692	Stressed ST692	
β-Lactams	Ampicillin	256	1024	4
	Amoxicillin	256	1024	4
	Cefalexin	32	64	2
	Cefazolin	32	64	2
	Cefoperazone	64	128	2
	Cefotaxime	32	32	1
	Cloxacillin	< 0.5	1	–^a^
	Imipenem	> 1024	> 1024	–^a^
	Methicillin	32	32	1
Chloramphenicol	Chloramphenicol	16	16	1
Aminoglycosides	Gentamicin	4	4	1
	Kanamycin	32	32	1
	Streptomycin	> 1024	> 1024	–^a^
Tetracyclines	Tetracycline	64	64	1

*MIC* minimum inhibitory concentration.

^*a*^Indicates not exactly known because the range of MIC is greater than the maximum value or less than a minimum value.

## Discussion

In this study, the potential function in β-lactam resistance of EVs released by bacteria in stressed conditions was examined. EVs from MRSA cultured with or without sub-MICs of ampicillin were purified, and their capability to degrade several β-lactam antibiotics investigated, and their proteomics compared to identified β-lactam antibiotic resistance-related protein constituents. It was found that the production of MRSA EVs increased dose-dependently by ampicillin treatment, and EVs from cultures treated with ampicillin became more potent in degrading β-lactam antibiotics.

Several factors that trigger vesicle production including genetic background of the strains, and factors such as temperature, iron and oxygen availability, media composition, and growth phase^10,22^. In addition, bacterial vesiculation could be triggered more intensely when bacteria were challenged with certain antibiotics at sub-lethal concentrations. A previous study showed that the treatment of β-lactam antibiotics creates holes in the peptidoglycan layer of Gram-positive bacteria which cause protrusion of cytoplasmic membrane material into the extracellular area and more EVs are generated^10,23–25^. There is no significant difference in the size of EVs isolated from MRSA treated w/ and w/o ampicillin by the analysis of TEM and DLS (Fig. 1 and Supplementary Table S1 in manuscript). However, the total protein amount of respective EVs was different in BCA protein assay. In addition, we have confirmed that the production of EVs augmented when ampicillin or gentamicin was treated with different MRSA strains named ST541 in sub-inhibitory concentration (data not shown).

Several types of research have shown that vesicles from bacteria can affect various antibiotics or antimicrobial peptides by hydrolysis/sequestration and acting as decoys. For instance, the vesicles can protect the bacteria for some antibiotics^16,24,26^ such as the case of colistin^18^, melittin^18^, polymyxin B^26^, and daptomycin^24^ thereby assisting the survival of the bacterium both in vitro and ex vivo^24^. It has been demonstrated in the past that vesicles can defense bacteria by degrading some β-lactam antibiotics, like amoxicillin, ampicillin, cefoperazone, and cefotaxime through their own proteins which are related to β-lactam resistance^7,9,19^. Packed into vesicles, substances related to antibiotic resistance can be delivered over long distances, unharmed by dilution and degradation^14^; such packaging poses a great benefit for the antibiotic resistance mechanism of the bacteria.

β-lactams are one of the most widely utilized groups of antibiotics available for the treatment of several bacterial infections^27^. β-lactam antibiotics attach to transpeptidase enzymes, also referred to penicillin-binding proteins (PBPs), interfering them from forming a peptidoglycan layer that produces the cell wall. Due to differences in cell wall organization, Gram-positive bacteria are bounded by a single membrane making them more sensitive to the bactericidal activity of β-lactam antibiotics than Gram-negative which are surrounded by an outer membrane that can protect exposure to antibiotics^28^. These bacteria have been exposed to naturally occurring antibiotic compounds for at least one billion years, and the evolutionary advantages they have adapted have led to the rapid development of resistance mechanisms for survival against antibiotics which are currently being used for medical applications^29^. The major mechanisms by which Gram-positive bacteria have developed to avoid the inhibitory effect of β-lactam antibiotics are as follows^30^: (1) β-lactamases are secreted into the extracellular space to degrade β-lactam antibiotics before they reach the cell wall; (2) Expression of mutated PBPs that are still capable of synthesizing the cell wall but are incapable of binding to β-lactam antibiotics. Therefore, EVs that have the ability to resist the bactericidal effect of β-lactam antibiotics as newly observed in Gram-positive strains are more important than those from the less susceptible Gram-negative strains.

The β-lactamase enzyme^7^ and metallo β-lactamase superfamily protein^31^ hydrolytically destroy β-lactam antibiotics and both proteins were significantly upregulated in EV_Strs_ compared with EV_Nor_ (Table 2 and see Dataset 1 and Dataset 2 online). It is assumed that the β-lactamase may be bound to the membrane of EVs^32^, therefore the EVs secreted from Gram-positive *S. aureus* exhibit β-lactamase activity (Fig. 8) and degrade antibiotics in the extracellular environment (Fig. 5). The number of PBPs located on the extracellular surface of the cytoplasmic membrane varies between bacterial species^33,34^. Cell wall synthesis in *S. aureus* is intrinsically controlled by four native PBPs, PBP1 to 4, and β-lactam resistance of MRSA is determined by the production of one non-native PBP, PBP2a^33^. Once β-lactam covalently bound to native PBPs, this stable covalent adduct could not be removed by neutral buffers, acids or detergents^35,36^, therefore we assumed that the native PBPs which are more abundant in EV_Strs_ than EV_Nor_ (Table 2) are capable of holding β-lactam antibiotics. This study hypothesized that EV_Strs_ are more efficient in degrading β-lactam antibiotics than EV_Nor_, and this capability can be due to the proteins produced in abundance since bacteria have a mechanism to selectively package-specific proteins and concentrate them into the vesicles to resist stressors^37,38^.

We confirmed that EVs did not affect the growth of strains in the presence of the other tested antibiotics (data not shown) which include imipenem, cefotaxime, and methicillin. These three antibiotics belong in the group of β-lactam antibiotics but were not degraded by the EVs containing β-lactamases due to their resistance to β-lactamase^39–41^. Therefore, although the activity of β-lactamase was increased fourfold by ampicillin stress in ST692 cells (Fig. 8c), these three mentioned antibiotics were not influenced in terms of their MICs by the stressed ST692 (Table 3).

Ampicillin below concentrations of MICs value intensifies the production of chromosomal β-lactamase^42,43^ and this phenomenon was confirmed in Fig. 8C and Table 3. These findings suggested that MRSA temporarily facilitates the intrinsic ability of bacterial defense mechanism to over-produce β-lactamase as a reaction after exposure to high levels of ampicillin drug, thereby causing an increased hyposensitivity to β-lactam antibiotics. Thus, for an appropriate antibiotic challenge, it might require an increased dose of antibiotics to treat bacteria.

Actually, *S. aureus* has been identified as one of the microbial infections in many polymicrobial infections^44^. Studies proving ‘cooperative interaction’ between different strains of bacteria had been discussed in the past. For example, *Candida albicans* and *S. aureus* colonized human mucosal surfaces with commensals and cause enhanced disease severity during biofilm-related coinfections^44–46^. *Haemophilus influenzae* and *S. aureus* showed cooperative interactions and both colonized in nasopharynx, instances, and genital tract^44,47^. *S. aureus* co-colonized together with *Enterococcus faecalis* in the intestinal tracts^44^, and conjugation between the two strains causes a horizontal transfer of the *vanA* gene, resulting in multidrug-resistant staphylococci^48,49^. Schaar et al. also indicated that vesicles including β-lactamase help to protect producer bacteria as well as co-occurring organisms in the human^19,50^. The results of Fig. 4 further imply that the EVs secreted from MRSA can indeed protect co-existing bacterial communities against β-lactam antibiotics.

To summarize, global protein modulation of EVs by ampicillin stress response of MRSA involves processes that are directly related to β-lactam antibiotic resistance. EVs naturally secreted from MRSA possess β-lactam-resistant proteins, which can help bacteria to survive in the antibiotic environment by hydrolyzing the capacity of antibiotics. Inducing stress on MRSA with a sub-lethal dose of ampicillin could stimulate production of EVs enhancing the ability to consume antibiotic compared with EVs released in no-stress condition. Therefore, proper drug treatment is necessary to impede the progeny of MRSA because indiscriminate abuse of β-lactam antibiotics may create an opportunity for bacteria to be more resistant to β-lactam antibiotics. Besides, this is a novel mechanism not related to PBP2a-based, the major resistance mechanism of β-lactam antibiotics of MRSA strains known up to this point. This information equips us with a new perspective on how to lessen (if not eradicate) the impact of multi-drug resistant bacteria which impose a grave threat to global public health.

## Methods

### Bacterial strains

Methicillin-resistant *Staphylococcus aureus* C-S03-S237 strain of ST692^51^ isolated from the chicken was provided from Animal and Plant Quarantine Agency, Korea and β-lactam-sensitive *S. aureus* ATCC29213 strain was purchased from ATCC. Luria–Bertani (LB; Oxoid) broth or tryptone soya agar (TSA; Oxoid, United Kingdom) were used to grow both cells at 37 °C. Ampicillin-sensitive bacteria, *Escherichia coli* RC85^9^, and *Edwardsiella tarda* ED45^52^ were cultured on TSA and *Salmonella* spp. Sal26B^53^ was incubated on brain heart infusion (BHI; Oxoid) agar at 37 °C.

### Determination of minimum inhibitory concentrations

Nine β-lactam antibiotics known to confer bactericidal effects by inhibiting cell wall biosynthesis, namely ampicillin, amoxicillin, cefalexin, cefazolin, cefoperazone, cefotaxime, cloxacillin, imipenem, and methicillin (Sigma-Aldrich, USA) and five other class antibiotics, such as chloramphenicol, gentamicin, kanamycin, streptomycin, and tetracycline (Sigma-Aldrich) were selected. The minimum inhibitory concentration (MIC) of each antimicrobial agent was determined in ST692 and ATCC29213 cells using the broth-dilution method in 96-well plates^9,54^ according to Clinical and Laboratory Standards Institute (CLSI) guidelines, except that cation-adjusted Muller Hinton broth was substituted with LB. The MIC values were measured from three independent experiments.

### EVs isolation and characterization

EVs from ST692 cells were purified from bacterial culture supernatant as described previously^7^, with some modifications. Briefly, when isolating EV_Nor_, the strain was cultured in nutrient broth (NB; Difco) without any antibiotic addition. To determine the change in the production of EVs, the ampicillin dose was treated at 1, 4, 16, or 64 μg/mL (EV_Strs_ means EVs treated with 64 μg/mL of ampicillin when bacteria are cultured). Each culture medium was centrifuged at 6,000×*g* for 15 min and concentrated by QuixStand Benchtop system (GE Healthcare, Sweden). Each supernatant was centrifuged at 150,000×*g* at 4 °C for 3 h. Further purification was performed by a continuous sucrose density gradient followed by ultracentrifugation. The final EV pellet was resuspended in 10 mM Tris–HCl (pH 8.0) (Biopure, Korea) and filtered through a 0.2 μm filter (Thermo Fisher Scientific, IL). The protein yields of EVs samples were measured using a Pierce BCA protein assay kit (Thermo Fisher Scientific, USA). Transmission electron microscopy (TEM) of EVs was performed as previously described^9^ using a Tecnai G2 Spirit Twin TEM system (FEI, USA). Dynamic light scattering (DLS) of EVs for particle size distribution and measurement of zeta potential was performed as described previously^9^ using a Nano ZS instrument (Malvern Instruments, Malvern, UK) and the Zetasizer software (version 7.11; Malvern Instruments).

### Proteome analysis by liquid chromatography combined with tandem mass spectrometry (LC–MS/MS)

Each of ST692 EVs was mixed with sample buffer and separated by Sodium dodecyl sulfate–polyacrylamide gel electrophoresis (SDS-PAGE) according to Laemmli’s method^55^ and in-gel digestion was performed as previously described^9^. Each obtained peptide mixture was resuspended in 0.1% (v/v) TFA and passed through an analytical column (Zorbax 300SB-C18 75 μm i.d. × 15 cm column; Agilent, Germany) via a trap column (Zorbax 300SB-C18 300 μm i.d. × 5 mm column; Agilent). The peptides were separated from acetonitrile gradient of buffer A (0.1% (v/v) formic acid in water) and buffer B (0.1% (v/v) formic acid in pure acetonitrile) at a constant flow rate of 0.2 μl/min using the Agilent 100 series nano HPLC system coupled on-line to a LTQ ion-trap mass spectrometer (Thermo Fisher Scientific). The gradient started linearly with 5% B and rose linearly to 40% B over 100 min, increased to 80% B over 1 min, and then increased to 80% B isocratically over 15 min. Full-scan mode (*m/z* 350–1600) was enabled and three MS/MS scans were performed with a 30-s dynamic exclusion option set for each survey MS scan.

### Quantitative protein profiling, statistics and database searching

Peptide peaks were detected with an average peak width of 1 min and matched with a mass accuracy of at least 0.6 Da. Differentially expressed proteins were defined to exhibit more than twofold or greater increase/decrease in comparable intensity or the complete appearance/disappearance of the spot. After alignment of the retention times of the chromatogram, normalization was carried out with the measured intensity distribution and the proteome was quantified with the peak intensity ratio. The MS/MS spectra of the peptide peaks were searched against a SwissProt uni_bacteria database or *Staphylococcus aureus* USA300 strain database using Mascot 2.3 (Matrix Science, London, UK). The obtained LC/MS data were analyzed with the DeCyder MS software (version 2.0; GE Healthcare, Uppsala, Sweden). For quantitative profiling, proteins identified by multiple peptides with significant Mascot scores were selected (*p* < 0.05).

### In silicon analysis of functional associations

Gene ontology (GO) terms such as biological process, cellular component, and molecular function are derived from differentially expressed proteins obtained using a software tool for researching annotations of proteins (STRAP) version 1.5 (Boston University School of Medicine, USA).

### Effect of EVs on the growth of bacteria in the presence of β-lactam antibiotics

The effects of EV_Nor_ and EV_Strs_ on the cytotoxicity of β-lactam antibiotics were monitored by assessing the growth curves of EVs-treated *Staphylococcus aureus* (ATCC29213) cells as previously described with slight modifications^9^. The following antibiotics were used at the growth-inhibiting concentrations: ampicillin, 40 μg/mL; cefoperazone, 8 μg/mL; cefazolin, 1.25 μg/mL; amoxicillin, 40 μg/mL; cefalexin, 4 μg/mL; and cloxacillin, 1.25 μg/mL. Cultured ATCC29213 cells were separately inoculated in a medium containing each antibiotic and 1, 5, 25 μg/mL of EV_Nor_ or EV_Strs_. To test whether EVs can affect different genera of bacteria, Gram-negative bacteria such as RC85, ED45, and Sal26B were cultured in LB, TSB, and BHI, respectively, and same concentrations of EV_Nor_ or EV_Strs_ were treated in the medium with respective cultured bacteria in the presence of 30 μg/mL of ampicillin. The bacterial growth curves at OD_600_ were recorded at 2 h intervals up to 12 h, and then at 12 h intervals up to 48 h or 12 h intervals for 96 h using an xMark microplate spectrophotometer (Bio-Rad). The bacterial cultures since the last measurement time were streaked on TSA with or without the respective same concentrations of antibiotics to test whether the EVs could confer resistance to antibiotic-susceptible bacteria. The samples collected at certain time points (ampicillin, 12 h; cefoperazone, 12 h; cefazolin, 24 h; amoxicillin, 36 h; cefalexin, 96 h; and cloxacillin, 48 h) and quantitative plate assays were carried out^9^. The count obtained in the absence of antibiotics was taken as 100%, and the corresponding counts in the presence of different concentrations EV_Nor_ or EV_Strs_ plus the respective β-lactam antibiotics were calculated. Colonies from each cultured sample (*n* = 5, colonies per sample) were randomly selected and identified by matrix-assisted laser desorption ionization-time of flight mass spectrometry (MALDI-TOF MS)^56^ to check for contamination.

### Measurement of antibiotic concentrations

To evaluate whether EVs could directly degrade β-lactam antibiotics, the effects of EV_Nor_ and EV_Strs_ on the concentrations of six antibiotics in a cell-free system were analyzed by liquid chromatography/electrospray ionization mass spectrometry (LC-ESI-QQQ-MS/MS; 6420 Triple Quad LC/MS; Agilent) as previously described with slight modifications^9^. One microgram per milliliter or 5 μg/mL of EV_Strs_ or 5 μg/mL of EV_Nor_ in PBS were mixed with ampicillin (40 μg/mL), cefoperazone (8 μg/mL), cefazolin (1.25 μg/mL), amoxicillin (40 μg/mL), cefalexin (4 μg/mL) or cloxacillin (1.25 μg/mL). Filtered PBS containing the respective antibiotics without EVs was used as a positive control. In addition, to compare the degradability of EV_Nor_ and EV_Strs_ against ampicillin, 5 μg/mL of each EVs was added to various concentrations of ampicillin. The concentrations of antibiotics were recorded at 3-h intervals for 6 h or at 24-h intervals for 48 h in triplicate.

### Quantification of β-lactamase activity

To test for differences in β-lactamase activity between whole-cell lysates, supernatants, and EVs from the stressed condition and normal condition, a colorimetric β-lactamase activity assay kit (BioVision, Canada) was used according to the manufacturer’s instructions. The assay involves the hydrolysis of nitrocefin which produces a colored product that is measured by spectrophotometry (OD_490_). A Bradford assay kit (Thermo Fisher Scientific) was used to determine the protein concentrations of samples. Equivalent concentrations of each sample were dispensed to the wells of a clear flat-bottomed 96-well, and the provided nitrocefin and buffer were added. The OD_490_ was immediately measured in kinetic mode.

### Changes in the effect of EVs on β-lactam antibiotics by β-lactamase inhibitor

The effects of the β-lactamase inhibitor, sulbactam (Abcam, United Kingdom), were investigated by growth curve experiments. The β-lactamase inhibitors were added at the previously reported and fixed concentrations^9^, the final concentration of 25 μg/mL was set in each case. Briefly, the growth curves of ATCC29213 treated with antibiotics (ampicillin, 40 μg/mL; cefoperazone, 8 μg/mL; cefazolin, 1.25 μg/mL; amoxicillin, 40 μg/mL; cefalexin, 4 μg/mL; or cloxacillin, 1.25 μg/mL) plus sulbactam were determined in the presence of 25 μg/mL EV_Strs_ or EV_Nor_.

### Statistical analysis

Statistical analyses were carried out using Graphpad Prism, version 8.1.1. (GraphPad, CA, USA). Significant differences were determined by Student’s *t*-test, One-way Analysis of Variance (ANOVA), Two-way ANOVA, or Turkey’s multiple comparison test. All data were expressed as means ± standard deviations (SD). Differences were considered statistically significant at *P* < 0.05.

## Supplementary information

Supplementary Information 1.

Supplementary Information 2.

Supplementary Information 3.

## Data Availability

All data generated or analyzed during this study are included in this published article and its supplemental material files.
