# Supplementary material for: Significant increase in the secretion of extracellular vesicles and antibiotics resistance from methicillin-resistant Staphylococcus aureus induced by ampicillin stress
Source: Sci Rep. 2020 Dec 3;10:21066. doi: 10.1038/s41598-020-78121-8 (PMC7713300; doi:10.1038/s41598-020-78121-8)
Supplement: Supplementary file 1 — Supplementary Information 1. [file 41598_2020_78121_MOESM1_ESM.docx]

**Significant increase in the secretion of extracellular vesicles and antibiotics resistance from methicillin-resistant *Staphylococcus aureus* induced by ampicillin stress**

Si Won Kim^1^, Jong-Su Seo^2^, Seong Bin Park^3^, Ae Rin Lee^1^, Jung Seok Lee^1^, Jae Wook Jung^1^, Jin Hong Chun^1^, Jassy Mary S. Lazarte^1^, Jaesung Kim^1^, Jong-Hwan Kim^2^, Jong-Wook Song^2^, Christopher Franco^4^, Wei Zhang^4^, Min Woo Ha^5^, Seung-Mann Paek^5^, Myunghwan Jung^6^, and Tae Sung Jung^1,4,*^

^1^Laboratory of Aquatic Animal Diseases, Institute of Animal Medicine, College of Veterinary Medicine, Gyeongsang National University, Jinju, 52828, Republic of Korea

^2^Environmental Chemistry Research Center, Korea Institute of Toxicology Gyeongnam Department of Environmental Toxicology and Chemistry, Jinju, 52834, Republic of Korea

^3^Coastal Research & Extension Center, Mississippi State University, MS 39567, United States

^4^Centre for Marine Bioproducts Development, College of Medicine and Public Health, Flinders University, Bedford Park, Adelaide, SA 5042, Australia

^5^College of Pharmacy and Research Institute of Pharmaceutical Sciences, Gyeongsang National University, Jinju, 52828, Republic of Korea

^6^Department of Microbiology, Research Institute of Life Sciences, College of Medicine, Gyeongsang National University Jinju, 52727, Republic of Korea

^*^Address correspondence to Tae Sung Jung, [jungts@gnu.ac.kr](mailto:jungts@gnu.ac.kr)

**SUPPLEMENTARY TABLE**

|  | Size (nm) | Polydispersity | Z-potential (mV) |
| --- | --- | --- | --- |
| EV_Strs_ | 78.22±0.81 | 0.259±0.009 | –37.73±0.85 |
| EV_Nor_ | 86.84±0.25 | 0.212±0.003 | –40.83±1.14 |

**Supplementary Table S1.** Physical characterization of EVs from stressed condition (EV_Strs_) and normal condition (EV_Nor_).

**SUPPLEMENTARY FIGURES**


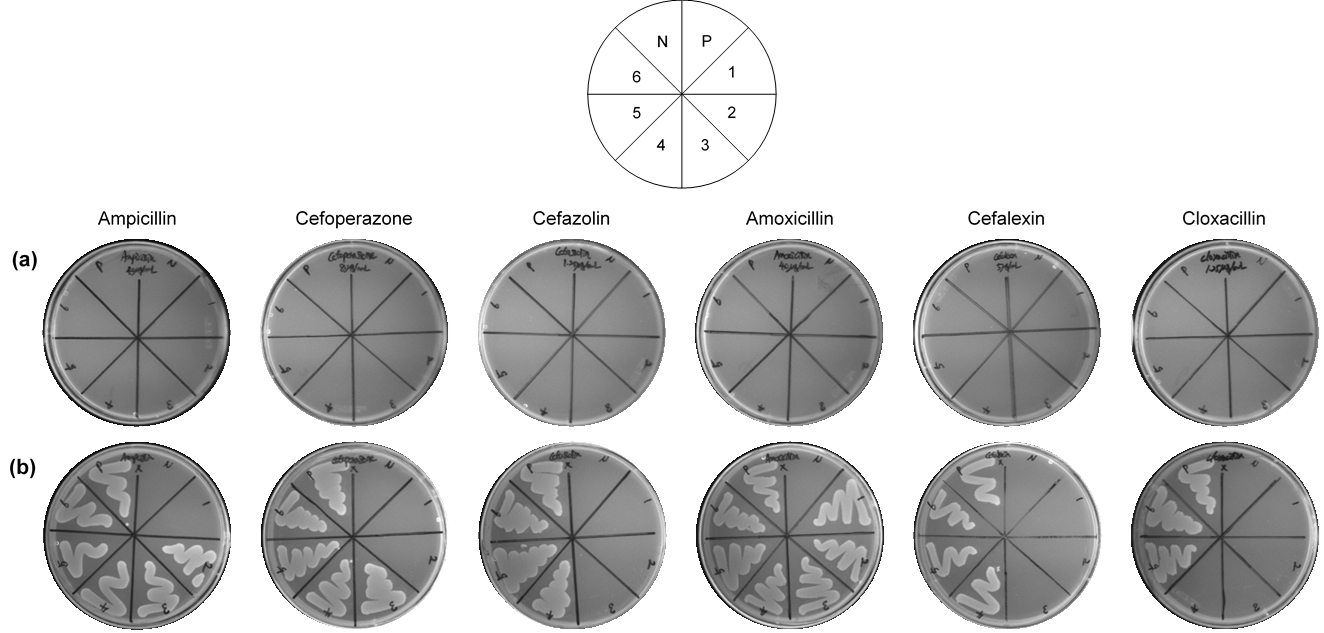


**Supplementary Figure S1.** Respect qualitative assays of bacterial samples from growth curve experiment on LB agar plates with presence of the antibiotics **(a)** or without them **(b)**. The growth-inhibiting concentrations of antibiotics were: ampicillin, 40 μg/mL; cefoperazone, 8 μg/mL; cefazolin, 1.25 μg/mL; amoxicillin, 40 μg/mL; cefalexin, 4 μg/mL; and cloxacillin, 1.25 μg/mL. Each labeling means: P, lb + ATCC29213; 1, antibiotic + ATCC29213 + EV_Nor_ 1 μg/mL; 2, antibiotic + ATCC29213 + EV_Nor_ 5 μg/mL; 3, antibiotic + ATCC29213 + EV_Nor_ 25 μg/mL; 4, antibiotic + ATCC29213 + EV_Strs_ 1 μg/mL; 5, antibiotic + ATCC29213 + EV_Strs_ 5 μg/mL; 6, antibiotic + ATCC29213 + EV_Strs_ 25 μg/mL; N, antibiotic + ATCC29213.


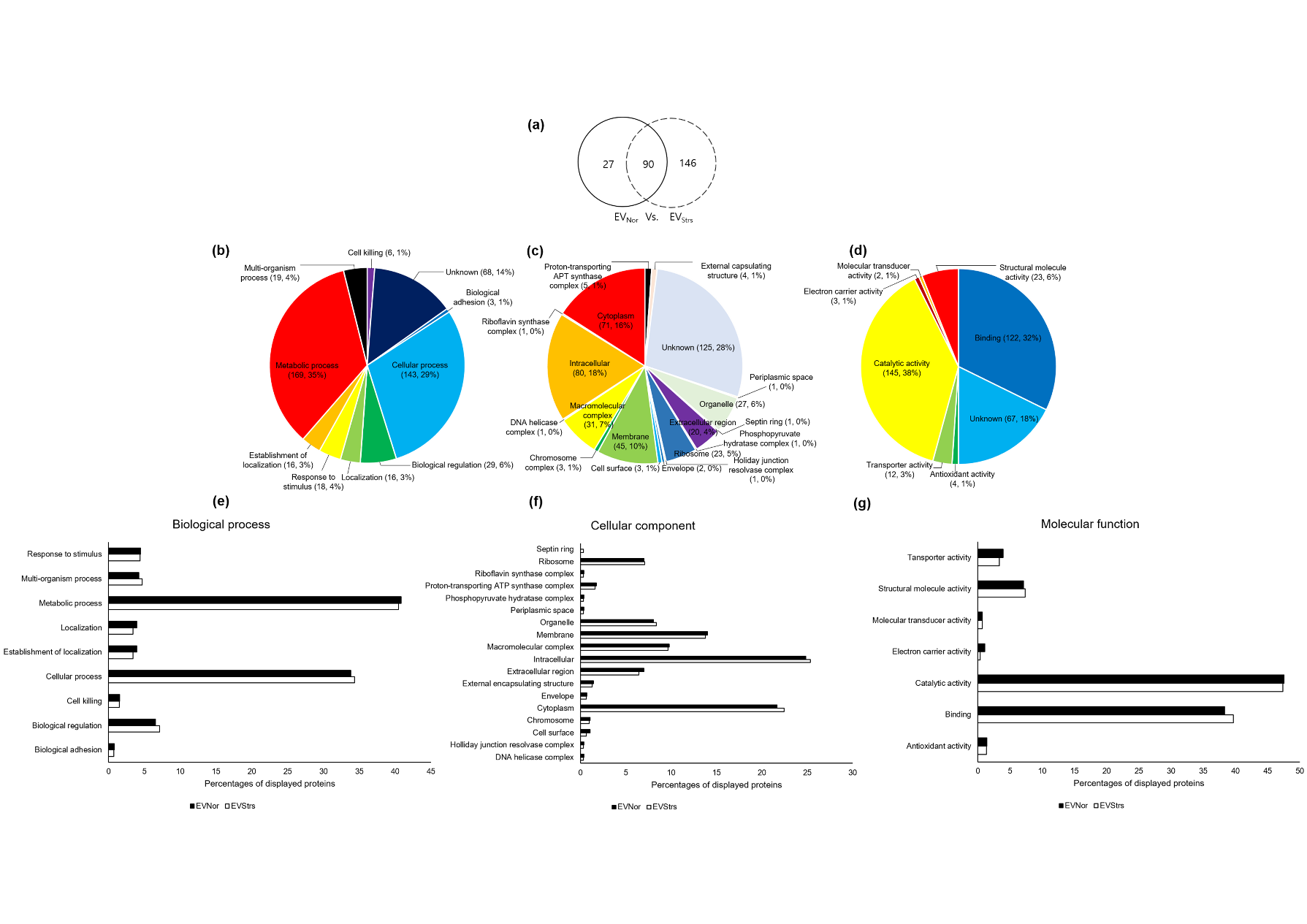


**Supplementary Figure S2.** Venn diagrams represent the total proteins achieved from EV_Nor_ and EV_Strs_ searched against the *Staphylococcus aureus* database while the bar graphs classify the proteins which showed differential expression in EV_Nor_ and EV_Strs_. **(a)** A total of 263 proteins were established in EV_Nor_ (117 Proteins) and EV_Strs_ (236 Proteins) together. These entire proteins were classified depending on the related biological process **(b)**, cellular component **(c)**, and molecular function **(d)**. The percentages of displayed EV_Nor_ (black) and EV_Strs_ (white) proteins were compared with respect to the consistent biological process **(e)**, cellular component **(f)**, and molecular function **(g)**.
